# Factors influencing death attitudes of medical students: a scoping review

**DOI:** 10.3389/fpubh.2024.1342800

**Published:** 2024-04-08

**Authors:** Jingjing Tong, Qian Liu, Ying Liu, Juan Li, Qin Zhang, Huashan Shi

**Affiliations:** ^1^Department of Postgraduate Students, West China Hospital/West China School of Medicine, Sichuan University, Chengdu, China; ^2^Innovation Center of Nursing Research and Nursing Key Laboratory of Sichuan Province, West China Hospital, Sichuan University/West China School of Nursing, Sichuan University, Chengdu, China; ^3^West China School of Nursing, Sichuan University, Chengdu, China; ^4^Department of Biotherapy, Cancer Center, West China Hospital, Sichuan University, Chengdu, China

**Keywords:** medical students, death attitude, death education, scoping review, mental health

## Abstract

**Aim:**

To summarize factors influencing death attitudes of medical students, help identify intervention targets, and design precision interventions for improving death attitudes of medical students.

**Methods:**

Web of Science, PubMed, Embase, OVID, China National Knowledge Infrastructure, and Wanfang databases were searched. Retrieval time was from January 2012 to September 2023. Studies on factors influencing death attitudes of medical students were included.

**Results:**

Thirty-five studies were included in the final review. A total of 28 factors influencing death attitudes of medical students were summarized and divided into three categories comprising personal factors, social factors, and psychological factors. More than 15 studies confirmed that gender, religion, and discussing death with families were factors that influenced medical students’ death attitudes.

**Conclusion:**

Results indicate that there are many types of factors that influence death attitudes of medical students. It is necessary for universities to implement death education based individual characteristics and guide medical students to cultivate generally optimistic death attitudes and appropriate life values.

## 1 Introduction

The number of medical students is quickly rising within the global health care system (1). However, a number of physical and mental health issues that have emerged during the medical students’ growth process have attracted considerable attention in recent years. Medical students belong to a group of individuals that experience relatively high pressure. Violence, homicide, suicide, and other malignant episodes have recently increased, which is indicative feelings of meaninglessness in the lives of medical students (2). Individual emotional and psychological feedback about their own death or the death of others has been researched for decades, with these attributes the subject of a 1936 study of death attitudes. As future medical professionals, medical students constantly come into contact with death. As a result, their outlooks on life and attitudes toward death influences their future employment potential and their ability to cope with death (3). It has been shown that medical students’ attitudes toward death have a dramatic impact on almost every aspect of their development, particularly their mental health and cognitive abilities (4), death education can assist medical students better comprehend the purpose and worth of life as well as help them have a positive outlook on death (5). Medical students, as future medical workers, will encounter death on a regular basis, and their attitudes toward death will have a direct impact on their personal growth as well as their attitudes and behaviors in the later stages of complex clinical work. Furthermore, medical students who receive death education can develop a stronger sense of social duty as well as become more adept at spreading the word about death education, which will benefit society as a whole (6, 7).

There is an urgent need for colleges to develop guidance for medical students in adopting appropriate views of death, to help them build a mature education guidance system, and to develop distinct education programs with different attributes that match individual characteristics. This paper summarizes the latest research progress on the factors that influence medical students’ death attitudes, and makes a reasonable analysis of these factors, aiming to provide new ideas and references for universities to set up death education systems.

## 2 Article types

The current article is a review. Specifically, the Joanna Briggs Institute methodology for scoping reviews (8) guided the current methodology. The primary review issue concerned the factors that influence medical students’ death attitudes.

## 3 Methods

### 3.1 Literature search strategy

We searched six electronic databases, namely Web of Science, PubMed, Embase, OVID, China National Knowledge Infrastructure, and Wanfang databases. Retrieval time was from January 2012 to September 2023. Boolean operators were used, with two librarians helping to develop the search strategy. The Boolean search combined subject and free words, including “medical student,” “nursing student,” “death attitude,” and “attitude toward death.”

### 3.2 Study selection

The following inclusion criteria for studies was applied: (1) research subjects were medical students, including clinical medicine students and nursing students; (2) study of factors influencing death attitudes of medical students; (3) full text available; and (4) articles published in Chinese or English. Exclusion criteria included: (1) intervention research on death attitudes of medical students and (2) books, editorials, letters, or conference abstracts. Searched studies were imported into NoteExpress to remove duplicate studies. Two reviewers independently selected the remaining studies by screening the title and abstract. If studies met inclusion criteria, the full text was screened, and studies that continued to meet inclusion criteria were explored further. Selection results were cross-checked, and a third reviewer was invited to discuss and make a final decision when there were differences.

### 3.3 Data extraction

Two reviewers independently screened the full text of each included article. Extracted data included authors, country, publication year, sex ratio, samples, and questionnaire used to investigate death attitudes. To ensure validity, two reviewers cross-checked the extracted data.

### 3.4 Data analysis

The extracted data were discussed by the team. We summarized the factors influencing death attitudes of medical students and divided the influencing factors into three categories comprising personal factors, social factors, and psychological factors.

## 4 Results

### 4.1 Characteristics of included studies

**Figure 1:**
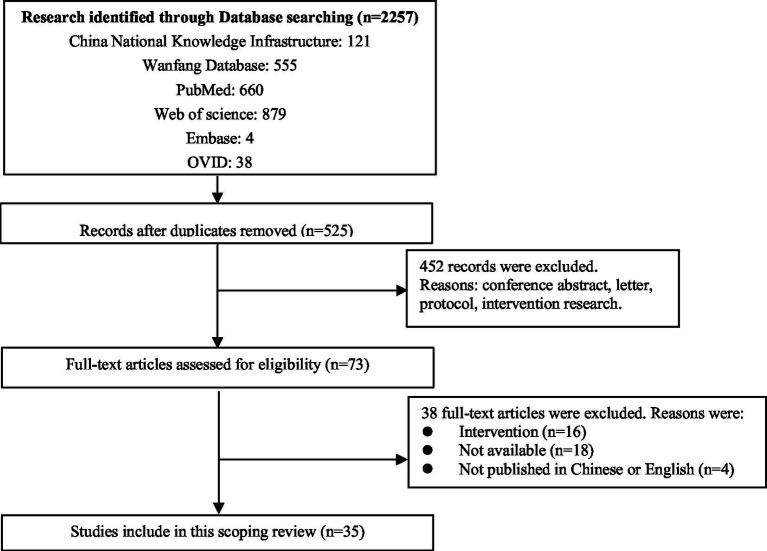
Flow diagram of study selections.

As shown in Figure 1, a total of 2,257 articles were initially retrieved, and 1,732 duplicates were removed. After screening the titles and abstracts, 452 records were removed because study type did not meet inclusion criteria (e.g., conference abstracts, letters, protocols, and intervention research). After screening 73 full texts, 38 studies were further excluded, of which 16 were intervention studies, 18 were not published in Chinese or English, and 18 did not have full text available. After all screening, a total of 35 studies were included in the current scoping review (9–44). As shown in Table 1, most of the included studies came from China, while seven studies came from Iran, Spain, Korea, Bolivia, Poland, and Palestine. Student majors included medicine, nursing, physiotherapy, pharmacy, rehabilitation, midwifery, medical laboratory technology, and preventive medicine. Additionally, most of the included studies used the Death Attitude Profile-Revised questionnaire to measure the death attitudes of medical students.

**Table 1:** General information of included studies.

Author, year	Country	Sample (Male/Female)	Major	Age	Questionnaire
Xie et al., 2014 (9)	China	757 (NR)	Medicine	19.6 ± 1.17	Death Attitude Profile-Revised
Liu et al., 2015 (10)	China	291 (93/198)	Medicine	17–25	Investigator design
Luo et al., 2016 (11)	China	235 (59/176)	Medicine and nursing	21.4 ± 0.86	Death Attitude Profile-Revised
Asadpour et al., 2016 (12)	Iran	308 (33/275)	Medicine	21.28 ± 2.16	Death Attitude Profile-Revised
Nong et al., 2017 (13)	China	1,062 (312/750)	Medicine and nursing	NR	Death Attitude Profile-Revised
Yang et al., 2018 (14)	China	430 (103/326)	Medicine and nursing	21. 68 ± 1. 72	Investigator design (Students’ basic personal information, exposure to mortality, and their attitudes regarding death were collected)
Nong et al., 2018 (15)	China	1,062 (312/750)	Medicine and nursing	NR	Death Attitude Profile-Revised
Pérez-de la Cruz S, et al., 2018 (16)	Spain	411 (312/279)	Medicine, nursing, and physiotherapy	23.3 (mean age)	The modified Bugen Scale for facing death
Chen et al., 2019 (17)	China	406 (141/265)	Medicine, public health, nursing, and health management	21.17 ± 1.46	Death Attitude Profile-Revised
Liu et al., 2019 (18)	China	730 (154/576)	Medicine and nursing	NR	Death Attitude Profile-Revised
Xu et al., 2019 (19)	China	366 (20/337)	Nursing	NR	Death Attitude Profile-Revised
Kim et al., 2019 (20)	Korea	184 (26/73)	Nursing	20.99 ± 1.34	Death Attitude Profile-Revised
Wang et al., 2020 (21)	China	300 (72/228)	Nursing	20–25	Death Attitude Profile-Revised
Hu et al., 2020 (22)	China	244 (59/185)	Medicine	NR	Death Attitude Profile-Revised
Perez-de la Cruz et al., 2020 (23)	Spain, Bolivia	548 (183/365)	Medical, nursing and physiotherapy	20.7 ± 2.7	Death Attitude Profile-Revised
Wu et al., 2021 (24)	China	435 (104/331)	Medicine and nursing	21.58 ± 1.43	Death Attitude Profile-Revised
Qian et al., 2021 (25)	China	166 (12/154)	Nursing	NR	Death Attitude Profile-Revised
Ke et al., 2021 (26)	China	956 (143/813)	Nursing, pharmacy, rehabilitation, and midwifery	18.90 ± 2.10	Death Attitude Profile-Revised
Gu et al., 2021 (27)	China	212 (NR)	Nursing	NR	Death Attitude Profile-Revised
Wang et al., 2021 (28)	China	603 (236/367)	Medicine	NR	Death Attitude Profile-Revised
Xu et al., 2021 (29)	China	430 (83/347)	Medicine, nursing, pharmacy, and medical laboratory technology	19.07 ± 1.10	Death Attitude Profile-Revised
Zhou et al., 2021 (30)	China	230 (NR)	Medicine, nursing, and preventive medicine	NR	Death Attitude Profile-Revised
Fan et al., 2021 (31)	China	726 (98/628)	Nursing	Nursing	Death Attitude Profile-Revised
Xie et al., 2021 (32)	China	2,119 (125/1994)	Nursing	17.60 ± 1.44	Death Attitude Profile-Revised
Feng et al., 2022 (33)	China	991 (NR)	Medicine	NR	Death Attitude Profile-Revised
Niu et al., 2022 (34)	China	608 (138/470)	Medicine and nursing	20.52 ± 0.8	Death Attitude Profile-Revised
Ni et al., 2022 (35)	China	200 (40/160)	Nursing	NR	Death Attitude Profile-Revised
Zdziarski et al., 2022 (36)	Poland, Palestine	309 (92/217)	Nursing	22.25 ± 3.35	Death Attitude Profile-Revised
Miranda-Chavez et al., 2022 (37)	Peru	284 (119/165)	Medicine	22 (20–24)	Death Attitude Profile-Revised
Zahran et al., 2022 (38)	Jordan	555 (262/293)	Nursing	21.0 ± 4.1	Death Attitude Profile-Revised
He et al., 2022 (39)	China	382 (219/163)	Medicine	25.47 ± 3.16	Death Attitude Profile-Revised
Yu et al., 2022 (40)	China	1,410 (227/1183)	Nursing	20.36 ± 1.64	Death Attitude Profile-Revised
Feng et al., 2022 (41)	China	991 (NR)	Medicine	20.07 ± 1.16	Death Attitude Profile-Revised
Su et al., 2023 (42)	China	200 (74/126)	Medicine	NR	Death Attitude Profile-Revised
Han et al., 2023 (44)	China	1,044 (NR)	Medicine and nursing	NR	Death Attitude Profile-Revised

NR, Not reported.

### 4.2 Personal factors related to death attitudes

#### 4.2.1 Gender

A total of 19 studies explored the relationship between gender and death attitudes of medical students (9, 10, 12, 14, 15, 17, 19–22, 24–26, 32, 34, 38, 39, 41, 42). As shown in Table 2, four studies indicated that females generally displayed higher scores on fear of death assessments in comparison to males (9, 12, 34, 39). Three studies showed that males exhibited greater scores on the death avoidance dimension when compared to females (15, 19, 25). In contrast, other studies arrived at different conclusions (26, 32). Furthermore, there were different findings in the approach acceptance dimension. Some scholars found that males exhibited greater scores than females on this particular dimension (25, 39), whereas Ke et al. and Chen et al. reported that females demonstrated higher scores on the acceptance dimension (17, 26). However, another study indicated that gender differences did not yield any noteworthy impact on the views of medical students toward death (22).

**Table 2:** Personal factors related to death attitudes of medical students.

Author, year	Gender	Age	Grade	Religion	Origin	Profession	Family status	Physical condition	Character	Race	Average monthly spending	University	Education level
Xie et al., 2014 (9)	√		√										
Liu et al., 2015 (10)	√			×	√				√				
Luo et al., 2016 (11)				×		√	×						
Asadpour et al., 2016 (12)	√	√	√				√						√
Nong et al., 2017 (13)						√		√					
Yang et al., 2018 (14)	√	√	√	√		√							
Nong et al., 2018 (15)	√		√			√		√					
Pérez-de la Cruz S, et al., 2018 (16)						√							
Chen et al., 2019 (17)	√			√	√		√						
Liu et al., 2019 (18)			√	√									
Xu et al., 2019 (19)	√	×		√			√						
Kim et al., 2019 (20)	√		√										
Wang et al., 2020 (21)	√		×	√	√		√						
Hu et al., 2020 (22)	×		×	√								×	
Perez-de la Cruz et al., 2020 (23)					×								
Wu et al., 2021 (24)	√												
Qian et al., 2021 (25)	√			√			√	√					
Ke et al., 2021 (26)	√		√			√	√	√					
Gu et al., 2021 (27)				√									
Wang et al., 2021 (28)				√			×				×		
Xu et al., 2021 (29)													×
Zhou et al., 2021 (30)			√			√							
Fan et al., 2021 (31)		√	√										
Xie et al., 2021 (32)	√				√			√					√
Niu et al., 2022 (34)	√		√	×	√	√							
Ni et al., 2022 (35)			√										
Zdziarski et al., 2022 (36)										√			
Miranda-Chavez et al., 2022 (37)		√		√									
Zahran et al., 2022 (38)	√	×	√									√	
He et al., 2022 (39)	√			√									
Feng et al., 2022 (41)	√			√							√		
Su et al., 2023 (42)	√	√											
Han et al., 2023 (44)		√		√									

√, statistically significant; ×, not statistically significant.

#### 4.2.2 Age

Eight studies explored the relationship between age and death attitudes of medical students (12, 14, 19, 31, 37, 38, 42, 44). In Table 2, Asadpour et al. (12) found an inverse relationship between age and the level of fear of death, indicating that individuals’ fear of death tends to diminish as they grow older. Zahran et al. (38) observed that age had no significant statistical impact on death attitudes, which was similar to the conclusion drawn by Xu et al. (19).

#### 4.2.3 Grade

A total of 14 studies explored the relationship between grade and death attitudes of medical students (9, 12, 14, 15, 18, 20–22, 26, 30–32, 34, 35, 38). As shown in Table 1, Kim et al. (20) observed that nursing students exhibited increasingly positive death attitudes as they progressed through higher grade levels. Similarly, another study discovered that students in advanced grades experienced higher levels of anxiety and fear of death than their lower-grade counterparts (18). Additionally, the results of a study conducted by Nong et al. (15) were similar, indicating that second-year college students scored significantly lower on the fear of death dimension in comparison to first-year college students. However, two studies found that grade was not an influencing factor of the death attitudes of medical students (21, 22).

#### 4.2.4 Religion

Sixteen studies have been conducted to assess the influence of religion on medical students’ death attitudes (10, 11, 14, 17, 19, 21, 22, 25, 27, 28, 34, 37, 39, 41, 44). As shown in Table 2, three studies observed a statistically significant distinction between scores of the approach acceptance dimension with and without religion (21, 25, 28). Medical students who held religious beliefs exhibited higher scores on the natural acceptance dimension (14, 22). Conversely, other studies found that religion did not have a significant impact on death attitudes among medical students (10, 11, 34).

#### 4.2.5 Origin

Seven studies looked at the impact of students’ origin on their attitudes toward death (10, 17, 21, 23, 32, 34, 41). Some studies from China have focused on differences in death attitudes among medical students from different origins, finding that urban medical students had a relatively more positive view of death than those from rural areas (10, 17, 21, 32, 34, 41). Additionally, Niu et al. (34) found that medical students from rural areas exhibited higher scores on the fear of death dimension, which was similar to another study (21). In contrast, Xie et al. (32) found opposite results. One study found that there were no significant differences between the two origins (23).

#### 4.2.6 Profession

Eight studies explored the relationship between profession and death attitudes of medical students (11, 13–16, 26, 30, 34). The findings of two studies indicated that nursing students exhibited a higher level of fear of death (11, 26). Nursing and midwifery majors exhibited a higher propensity than pharmacy and rehabilitation majors in terms of both approach acceptance and escape acceptance (26). In the realm of the natural acceptance dimension, one study reported that individuals pursuing clinical majors demonstrated higher scores (13), whereas another study arrived at a contrasting conclusion (34).

#### 4.2.7 Family status

As shown in Table 2, eight studies explored the relationship between family status and death attitudes of medical students (11, 12, 17, 19, 21, 25, 26, 28). Ke et al. (26) discovered that medical students with a large family size, including parents and three or more generations, showed lower scores in terms of acceptance methods and avoidance of acceptance. There was a significant difference in scores of the avoiding death dimension between medical students who were only children and medical students who had brothers and sisters (17, 25).

#### 4.2.8 Other personal factors

As shown in Table 2, there were some other personal factors related to death attitudes. Five studies explored the relationship between physical condition and death attitudes of medical students (13, 15, 25, 26, 32). Medical students who perceived themselves to be in better physical shape, or were actually in better physical shape, exhibited lower levels of fear of death and death avoidance (13, 26). Some studies have explored the correlation between average monthly expenditure (28, 41), university (22, 38), education level (12, 29, 32), and death attitudes of medical students; however, the results of these studies differed. Comprehensive education has been shown to be more likely to foster reasonably positive views toward death among medical students (12, 32). Only one study examined the effects of character (10) and race (36).

### 4.3 Social factors related to death attitudes

#### 4.3.1 Death education

As shown in Table 3, nine studies explored the relationship between death education and death attitudes in medical students. All of these, studies suggested that medical students who received death education demonstrated more positive views toward death (14, 20–22, 27–29, 31, 41). In particular, there was a significant decrease in scores on the dimension of fear of death (22, 27, 29, 31). In addition, Xu et al. (19) discovered that medical students who received death education showed a greater propensity for natural acceptance of death.

**Table 3:** Social factors related to death attitudes of medical students.

Author, year	Death education	Discussing death with families	Interaction with the scene of death	Experience of losing friends or family	Reading death-related books	Seeing death-related media	First-hand experience in caring for end-of-life patients	First-hand experience in caring for suicidal patients
Xie et al., 2014 (9)		√						
Liu et al., 2015 (10)			×					√
Luo et al., 2016 (11)		√	×					
Asadpour et al., 2016 (12)			×					
Nong et al., 2017 (13)		√						
Yang et al., 2018 (14)	√	√	√		√		√	
Nong et al., 2018 (15)		√	√					
Chen et al., 2019 (17)				√			√	
Liu et al., 2019 (18)				√				
Xu et al., 2019 (19)							√	
Kim et al., 2019 (20)	√							
Wang et al., 2020 (21)	√		√					
Hu et al., 2020 (22)	√	√	√					
Wu et al., 2021 (24)		√			√			
Qian et al., 2021 (25)		√	×		×	√		
Ke et al., 2021 (26)		√		√			√	
Gu et al., 2021 (27)	√	√		√				√
Wang et al., 2021 (28)	√	√	×	√				
Xu et al., 2021 (29)	√	√						
Fan et al., 2021 (31)	√							
Niu et al., 2022 (34)		√	×					
He et al., 2022 (39)		√	√					
Feng et al., 2022 (41)	√							
Su et al., 2023 (42)		√						
Han et al., 2023 (44)			√					

√, statistically significant; ×, not statistically significant.

#### 4.3.2 Discussing death with families

As shown in Table 3, 15 studies explored the relationship between talking about death with families and the death attitudes of medical students (9, 11, 13–15, 22, 24–29, 34, 39, 42), and various family death discussion styles significantly influenced medical students’ death attitudes. Xie et al. (9) discovered that this kind of conversation considerably decreased the fear of death in medical students, which was similar to the findings of three other studies (11, 26, 39). Some studies found that engaging in this particular form of discourse had a notable impact on diminishing scores related to the dimension of death avoidance (11, 26, 27). Furthermore, whether the family engaged in conversations about death may also have an impact on the scores of medical students in relation to the dimension of natural acceptance (42).

#### 4.3.3 Funeral experiences

As shown in Table 3, 12 studies explored the relationship between funeral experiences and the death attitudes of medical students (10–12, 14, 15, 21, 22, 25, 28, 34, 39, 44). Six studies found that these experiences did not significantly affect attitudes toward death (10, 12, 25, 28, 34). Two studies revealed that students who participated in funeral ceremonies exhibited more positive attitudes toward death (21, 44). In contrast, Hu et al. (22) discovered that medical students who had participated in funerals exhibited lower scores on the natural acceptance component than those who had not attended funerals.

#### 4.3.4 Experience of losing friends or family

As shown in Table 3, five studies explored the relationship between the experience of losing friends or family and their death attitudes (17, 18, 26–28). Ke et al. (26) discovered that the death of family members had a notable impact on medical students’ scores in the natural acceptance dimension, leading to a significant decrease. Two studies suggested that this situation caused an increase in medical students’ scores in the death avoidance dimension (27, 28).

#### 4.3.5 Other social factors

As shown in Table 3, there were some other social factors related to death attitudes. Reading death-related books (14, 24, 25), seeing death-related media reports (25), and having first-hand experience in caring for end-of-life (14, 17, 19, 26) or suicidal patients (10, 27) were factors that influenced death attitudes among medical students.

### 4.4 Psychological factors of death attitudes

As shown in Table 4, psychological well-being (20), sense of meaning in life (29, 40), suicidal thoughts (9), powerful emotional reaction to death (42), professional recognition (26, 32), and Internet addiction (33, 41) were factors that influenced death attitudes of medical students. Students who possessed a heightened feeling of spiritual well-being and a deep understanding of the meaning of life tended to exhibit a more distinct purpose in life and a greater appreciation for the worth of their own existence (20, 29, 40). Xie et al. (32) discovered a correlation between medical students’ level of professional recognition and their anxiety about death, which was similar to the results of another study (26). Additionally, two studies (33, 41) found that the degree of Internet addiction could influence medical students’ death attitudes.

**Table 4:** Psychological factors of death attitudes of medical students.

Author, year	Psychological well-being	Sense of meaning in life	Suicidal thoughts	Powerful emotional reaction toward death	Professional recognition	Internet addiction
Xie et al., 2014 (9)			√			
Kim et al., 2019 (20)	√					
Ke et al., 2021 (26)					√	
Xu et al., 2021 (29)		√				
Xie et al., 2021 (32)					√	
Feng et al., 2022 (33)						√
Yu et al., 2022 (40)		√				
Feng et al., 2022 (41)						√
Su et al., 2023 (42)				√		

√, statistically significant.

## 5 Discussion

Through the examination of the current studies, we determined that medical students’ attitudes toward death are influenced, to some degree, by individual characteristics, social interactions, and mental status. Colleges and institutions should consider the unique circumstances of medical students to implement death education in a focused and tailored manner.

### 5.1 Personal factors

Research examining the impact of gender on death attitudes revealed that females exhibit more fear of death than males, which is similar to the results of a previous review (43). This is likely due to the female tendency to engage in more emotional thinking and experience higher levels of anxiety, which consequently intensifies their fear of death (12, 39). Moreover, the contrasting attitudes toward death in clinical and nursing professions can be attributed to the higher number of females in the nursing field. This finding is similar to a previous study, which suggested that the nursing interns had statistically significantly higher scores in death attitudes compared to the norms (19). In addition, the nursing profession directly engages in patient care, which suggests that its curriculum and clinical experiences may influence its perspectives on death. However, additional comparative research is needed to confirm these findings.

Diverse researchers have reached varying conclusions regarding the disparities in death attitudes among medical students of different ages. This discrepancy can be attributed to the fact that the medical student population consists of individuals who are relatively similar in age, thereby limiting the ability to accurately assess the distinct impact of age on death attitudes. Study findings indicated that those with a longer duration of medical education, namely, those who were in higher grades, tend to exhibit more positive death attitudes compared to individuals in lower grades (18, 20). The hypothesis suggests that the primary factor behind this phenomenon is the progressive development of more positive attitudes toward death as individuals advance in grade level, which can be attributed to their exposure to clinical training in hospice care or education connected to death (20).

The perspectives of family members toward death, as well as familial backgrounds, exert a certain degree of influence on the perceptions of medical students regarding death. This finding is similar to the results of a previous review (43). The presence of a familial environment that encourages open acknowledgment and discourse about death, along with a strong sense of kinship, has the potential to assist medical students in mitigating their fear of death to a certain degree and fostering a more optimistic outlook on the subject of death (11, 21, 29, 39). If there are older adults within the family, the younger generation will inevitably encounter disease and death at a younger age, thus gradually forming a more accurate perception of death (26).

Various religions hold distinct notions regarding life and death, and it may be argued that religious convictions will influence individuals’ perspectives on death to some degree (21, 34). These results are similar to the previous study (43). Based on the analyzed studies, it is evident that the presence or absence of religious beliefs does not significantly influence medical students’ attitudes toward death. It is worth noting that some of these studies had limited sample sizes and lacked precise screening regarding the definition of, and loyalty toward, religious beliefs, which may have impacted the results. Further research is needed to thoroughly investigate this factor (34, 44).

Various countries exhibit distinct societies and cultures. However, this study lacks sufficient literature pertaining to race or country of origin, necessitating further investigation. Chinese scholars have conducted studies examining disparities in death attitudes between medical students from urban and rural backgrounds. The research suggests that an urban upbringing and higher levels of parental education may contribute to greater emphasis on the psychological development of children, resulting in a relatively positive outlook on death (10, 34).

In summary, while implementing death education, colleges and universities should tailor their approach based on several factors, including gender, location of origin, and family background. For instance, when targeting women, the emphasis should be on alleviating their apprehension toward death. Medical students hailing from rural areas should receive intentional guidance to approach death with a positive mindset. Death education should be conducted promptly and efficiently. Furthermore, it has been discovered that both family and society exert significant effects on the attitudes of medical students regarding death. Consequently, death education for medical students should extend beyond the confines of the school classroom. Schools should actively collaborate with families and communities of medical students to implement death education, aimed at assisting medical students in developing a positive attitude toward death through subtle impacts.

### 5.2 Social factors

The results of this study found that receiving death education can also improve medical students’ attitudes toward death. This is similar to results of previous studies (45, 46). It is evident that current universities are actively broadening their methods of death education and constructing effective death education classrooms, which can assist medical students in developing a more optimistic perspective on death. However, there are currently a limited number of universities that have truly integrated death education into their academic programs (20).

Based on the current findings, the impact of funeral experiences on medical students’ perspectives regarding death remains uncertain. It is necessary to conduct additional research to determine whether funerals, as a temporary situation involving direct confrontation with death, can aid medical students in developing favorable attitudes toward death. Experiencing the death of family members in life can result in medical students having negative attitudes toward death for a certain period of time.

In summary, death education can extend beyond conventional lecture formats, and confronting death can prove effective in enhancing medical students’ disposition toward death. Universities should actively engage in the exploration and discovery of comprehensive and varied approaches to death education. Simultaneous attention should be paid to the social experiences of medical students. This includes providing immediate support to students who have recently lost a family member to assist them to quickly adjust their physical and mental condition during critical moments. Further, universities should offer guidance to help students develop a constructive mindset and approach toward death through real-life encounters.

### 5.3 Psychological factors

Psychological well-being among medical students is a significant indicator of attitudes toward death. Students with relatively sound mental states tended to have more positive attitudes toward death. Psychological well-being is essential for medical students to effectively cope with the demanding circumstances they encounter, including the emotional toll of witnessing deaths in their professional setting and clinical training (20). Therefore, when implementing death education, universities should promptly consider the mental well-being of medical students and conduct customized death education based on their mental health. Additionally, there is a need to enhance the management system of universities to prevent medical students from developing Internet addiction, which consequently impacts their physical and mental well-being, as well as their attitudes toward death.

Furthermore, considering the distinctive characteristics of medical students, it is imperative to include professionalism education in conjunction with death instruction. By doing so, these two components can mutually reinforce each other, fostering the development of medical professionals who possess a scientific and professional approach toward death and who are also inclined to volunteer.

## 6 Limitations

In the current review, only six databases were searched, making it possible that some relevant studies may not have been included. Additionally, several studies written in languages other than Chinese and English. Furthermore, the studies identified primarily concentrated within Asia and surrounding regions, more pertinent studies have been conducted in China, necessitating greater investigation into the determinants that impact the attitudes of medical students toward mortality in other locations. Subsequent research will examine the global context in more detail.

## 7 Conclusion

Medical students’ perspectives on mortality are shaped by individual aspects, social experiences, mental status, and various other elements. Alterations in their perspectives about death can similarly impact their approach to patient care and end-of-life support. Universities should prioritize this issue and consider individual characteristics. They should implement focused death education programs to guide medical students in developing positive attitudes toward death and accurate life values. Additionally, they should ensure that students understand the psychological and physiological conditions and needs of dying individuals. Together, these interventions will improve the standard of care given to individuals with terminal illnesses and their families.

## Author contributions

JT: Conceptualization, Data curation, Methodology, Writing – original draft. QL: Data curation, Writing – review & editing. YL: Data curation, Writing – review & editing. JL: Writing – review & editing. QZ: Resources, Supervision, Writing – review & editing. HS: Supervision, Writing – review & editing.
